# Humanization of *Drosophila* Gαo to Model *GNAO1* Paediatric Encephalopathies

**DOI:** 10.3390/biomedicines8100395

**Published:** 2020-10-06

**Authors:** Mikhail Savitsky, Gonzalo P. Solis, Mikhail Kryuchkov, Vladimir L. Katanaev

**Affiliations:** 1 Translational Research Center in Oncohaematology, Department of Cell Physiology and Metabolism, Faculty of Medicine, University of Geneva, 1211 Geneva, Switzerland; mikhail.savitskiy@unige.ch (M.S.); gonzalo.solis@unige.ch (G.P.S.); Mikhail.Kryuchkov@unige.ch (M.K.); 2 School of Biomedicine, Far Eastern Federal University, 690690 Vladivostok, Russia

**Keywords:** paediatric encephalopathy, GNAO1, G protein, humanization, Drosophila, disease model

## Abstract

Several hundred genes have been identified to contribute to epilepsy—the disease affecting 65 million people worldwide. One of these genes is *GNAO1* encoding Gαo, the major neuronal α-subunit of heterotrimeric G proteins. An avalanche of dominant de novo mutations in *GNAO1* have been recently described in paediatric epileptic patients, suffering, in addition to epilepsy, from motor dysfunction and developmental delay. Although occurring in amino acids conserved from humans to *Drosophila*, these mutations and their functional consequences have only been poorly analysed at the biochemical or neuronal levels. Adequate animal models to study the molecular aetiology of *GNAO1* encephalopathies have also so far been lacking. As the first step towards modeling the disease in *Drosophila*, we here describe the humanization of the *Gαo* locus in the fruit fly. A two-step CRISPR/Cas9-mediated replacement was conducted, first substituting the coding exons 2–3 of *Gαo* with respective human *GNAO1* sequences. At the next step, the remaining exons 4–7 were similarly replaced, keeping intact the gene *Cyp49a1* embedded in between, as well as the non-coding exons, exon 1 and the surrounding regulatory sequences. The resulting flies, homozygous for the humanized *GNAO1* loci, are viable and fertile without any visible phenotypes; their body weight, locomotion, and longevity are also normal. Human Gαo-specific antibodies confirm the endogenous-level expression of the humanized Gαo, which fully replaces the *Drosophila* functions. The genetic model we established will make it easy to incorporate encephalopathic *GNAO1* mutations and will permit intensive investigations into the molecular aetiology of the human disease through the powerful toolkit of *Drosophila* genetics.

## 1. Introduction

Epilepsy is a chronic disease of multigenic origin, characterized by appearance, mostly in an unpredicted manner, of seizures, and sometimes complicated by other neurological or neurodevelopmental deficits [1]. Seizures result from an imbalance between the inhibitory and excitatory conductance in the brain and can be induced by acute toxic or traumatic impacts. The causes of the episodic shifts in the balance of inhibition and excitation seen in the chronic epilepsy remain largely unexplained. With 65 millions of people worldwide currently suffering from epilepsy and the inadequacy of the current pharmacological approaches to certain subtypes of the disease, the need to advance our understanding of the aetiology of this disease is clear, as is the urgency to develop novel medical treatments [2].

**Figure 1:**
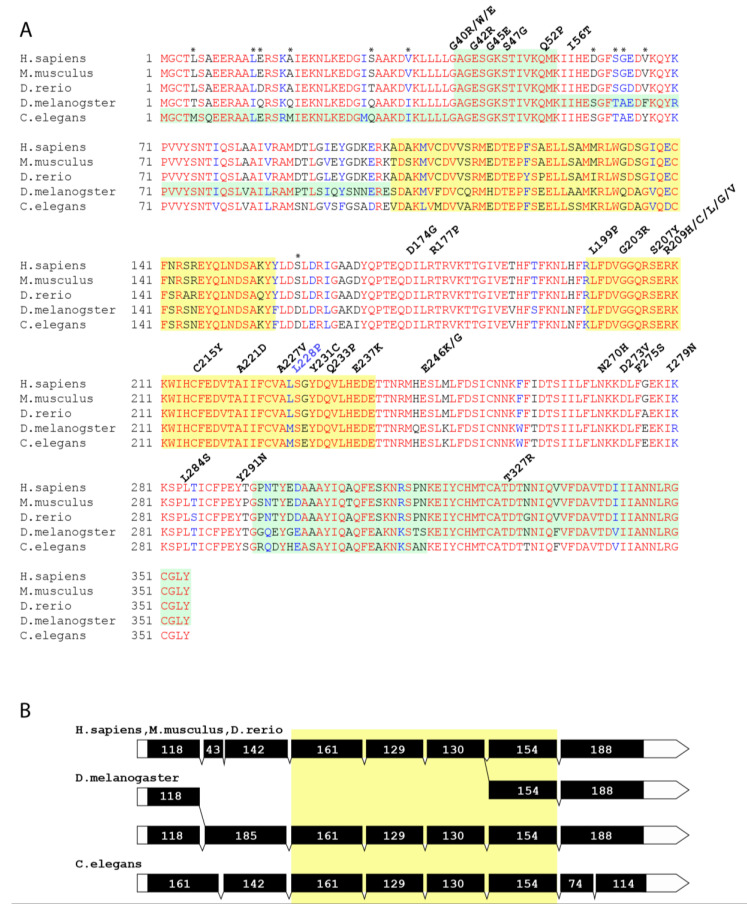
(**A**) Alignment of Gαo homologs from three vertebrate and two invertebrate organisms. Pale green and yellow colours on the background of the alignment mark the exon boundaries. Above the alignment, the known mutations associated with human neurological phenotypes are designated. The mutated amino acids belong to conservative parts of proteins (letter blocks of red). The mismatching amino acids that were not replaced during the editing of dGαo are designated with asterisks. For *Drosophila*, the dGαo-B isoform is shown. (**B**) Schematic exon-intron structures of Gαo homologs. Black boxes mark the coding parts of the exons, and white boxes indicate the noncoding parts. The drawing of the exons is presented in scale. Digits inside the exons mark their precise length in base pairs. The yellow box marks identical exons in the species compared.

Several hundred genes linked to epilepsy have been identified [3], and the estimate tells that this number will increase by folds, matching the number of genes linked to cancer and exceeding the diversity of the cancer-related genes by biological functions [4,5]. Advances have been made in the understanding of the epileptic molecular and cellular mechanisms and pathways (involving ion channels and their regulators, but also neuronal migration, synaptic plasticity, and neurite outgrowth, among other things [1,4,6]). However, many of the epileptic mutations remain enigmatic—both in the sense of how they provoke the disease, as well as in the sense of the broader molecular pathway, hijacked in epilepsy, of which they are part. One of the proteins that have recently emerged as an important player in epilepsy is Gαo—the major neuronal α-subunit of heterotrimeric G proteins, encoded by the gene *GNAO1*. Starting from 2013 [7], whole-exome sequencing in a subset of epileptic patients has resulted in an avalanche of described mutations in *GNAO1* (see [8] for the most up to date review). This subset represents paediatric patients with the early onset epileptic encephalopathy and Ohtahara syndrome, suffering additionally from movement disorders and developmental delays. These patients are resistant to conventional antiepileptic pharmacological treatments and may die early in life. A number of sites in Gαo have been found mutated in these epileptic patients, all affecting amino acids conserved among Gαo and its orthologues all the way down to fruit flies and nematodes (Figure 1A), highlighting the importance of the affected amino acids for the Gαo function. *GNAO1* mutations in epilepsy are heterozygous, suggesting their dominant nature. Interestingly, the relative degree of manifestation of different deficits (seizures, developmental delay, movement disorders) may be different depending on the exact amino acid mutation in Gαo [9,10,11]. Recently, a point mutation in Gαo was also identified to cause a severe childhood speech disorder [12].

Heterotrimeric G proteins are key signalling molecules best recognized as the immediate transducers of GPCRs (G-protein-coupled receptors). Coupled to GPCRs, heterotrimeric G proteins transduce signals from a large variety of extracellular cues, from quanta of light, ions, small organic molecules, to large macromolecules [13]. A heterotrimeric G protein complex consists of three subunits, α, β, and γ, of which the α-subunit is responsible for binding to guanine nucleotides as well as to the cognate GPCR. Sixteen vertebrate Gα subunits are classified into four major families based on sequence homology: Gαi/o, Gαs, Gαq and Gα12 [14]. Gαo belongs to the first family and transduces the signal of a group of rhodopsin-like GPCRs, including the opioid, α2-adrenergic, D2 dopaminergic, M2 muscarinic, and somatostatin receptors [15]. Gαo was among the first α-subunits discovered [16] and found to be the major Gα subunit of the central nervous system (CNS) across the animal kingdom [17,18], controlling both development and adult physiology of the brain [19,20]. Gαo knockout (KO) mice showed a strong developmental delay during the first 3 weeks after birth and a short half-life of only 7 weeks on average [20]. Gαo KO mice also presented multiple neurological abnormalities such as hyperalgesia, hyperactivity, generalized tremor with occasional seizures, and a severe impairment of motor control [19,20]. At the cellular level, dorsal root ganglion cells derived from Gαo KO mice presented a reduced inhibition of Ca^2+^ channel currents by the agonist-induced activation of opioid receptors [20]. Gαo has also been implicated in the regulation of Ca^2+^ and K^+^ channels in sensory and hippocampal neurons [19,21]. In addition to neurons, Gαo is expressed in the heart [22], where it is necessary for proper development and functioning of the organ [23,24]. Gαo has additional developmental functions such as cell fate determination and polarization, in part via the Wnt-Frizzled pathway [25,26], as well as pathological implications, e.g., in cancer [27,28].

Gαo expression was found to strongly increase during early neonatal rat brain development [29], where it is enriched in growth cones [30]. In the rat pheochromocytoma cell line PC12, Gαo expression rises during the process of neurite outgrowth induced by nerve growth factor (NGF) [31,32,33], and overexpression of a Gαo active mutant potentiates the effects of NGF in neurite length [34]. Similar to its mammalian counterpart, insect Gαo is strongly expressed in the CNS of the adult *Drosophila* [18]. Gαo transcript and protein were present at all stages of embryonic development with a marked increase during the period of active axonogenesis [35,36]. These early data suggested that Gαo may be involved in neuronal differentiation, a role supported by later studies showing reduced neurogenesis and increased cell death in the olfactory bulb of Gαo KO mice [37] and defects in guidance and axonal growth of motoneurons in Gαo mutant fruit flies [23]. Additionally, differentiation of mouse embryonic stem cells into dopaminergic neurons pointed to Gαo as one of the genes specifically upregulated during neurogenesis [38]. 

Driven by the pivotal roles of Gαo and by the fact that the list of its molecular targets was remarkably short [39], we have performed massive whole genome/proteome screenings to identify novel Gαo interaction partners. This analysis identified >250 proteins as novel candidate Gαo partners. “Cherry-picking” of individual proteins from this list resulted in detailed descriptions of novel mechanisms of Gαo-controlled regulation of Wnt/Frizzled signalling, neuromuscular junction formation, planar cell polarity, asymmetric cell divisions, etc. [40,41,42,43,44,45]. As opposed to such characterizations of selected individual Gαo partners, we next aimed at identifying functional modules within the Gαo interactome. Several functional modules (such as cytoskeleton organization, cell division, cell adhesion, etc.) were identified within the Gαo interactome; subsequent work identified Gαo as a master regulator of vesicular trafficking [46]. Remarkably, across different cell types (neuronal, mesenchymal, epithelial) and species (insects and mammals), Gαo is found to dually localize to PM (plasma membrane) and Golgi [46,47]. This dual localization is found to play a coordinated role in the formation of cellular protrusions (such as neurites in neuronal cells), so that the PM pool of Gαo entices the initiation of the protrusion, while the Golgi pool ensures material delivery to it, permitting its elongation and stabilization. In the nervous system, these novel Gαo activities are necessary not only for neuritogenesis, but also for synaptogenesis [46]. As improper synaptic plasticity and process outgrowth are both implicated in the aetiology of epilepsy [1], the possible dominant effects of the epileptic Gαo mutations on these novel Golgi-emanating mechanisms discovered by us need to be investigated in detail, as are the molecular events happening at PM. Furthermore, with several established epileptic mutations affecting proteins working in Golgi-mediated trafficking (EpilepsyGene. Available online: http://www.wzgenomics.cn/EpilepsyGene/ (accessed on 05.10.2020)), and likely more to emerge, such an investigation could be the entry point into the identification of a novel epileptic pathway, involving Gαo-mediated trafficking routes. 

Of the point mutations identified within the coding region of *GNAO1* in paediatric encephalopathy patients, all correspond to highly conserved residues (Figure 1A), indicating their involvement in basic Gαo functions. However, the molecular characterization of these mutants has so far been very limited and provides somewhat contradicting findings on the mutant protein expression in heterologous cells, the effects of the mutants on basal and norepinephrine-induced N-type calcium channels, and on the forskolin-stimulated cAMP production [7,48]. Thus, the molecular mechanism(s) of Gαo mutants in the development of the severe neurological disorders await the much-needed detailed clarification to reveal potential therapeutic targeting approaches.

Animal models have the instrumental role in deciphering the disease mechanisms and in identifying/validating the treatment routes. *Drosophila* has been used to model a variety of human maladies, especially neurological ones [49,50,51,52,53,54]. As the first step towards modeling *GNAO1* paediatric encephalopathies in the fruit fly, we here describe the humanization of the *Drosophila* Gαo locus, finding the human protein fully replaces the insect one’s functions without any aberrations.

## 2. Materials and Methods

### 2.1. Plasmids

#### 2.1.1. Donor Plasmid pLdhGao23R for the First Round of Humanization dGαo

Synthesis of the DNA fragment of humanized *Drosophila Gαo* containing exons 2 and 3 with the intron between them was ordered from Synbio Technologies (Monmouth Junction, NJ, USA) as the plasmid pUC57-dhGαo23. The synthesized fragment contained nucleotide substitutions in the *Drosophila* sequence minimally needed to encode the human amino acids. Additionally, synonymous substitutions were introduced in the target sites for the gRNA-Cas9 complex in order to avoid double-strand breaks. The fragment was further designed to contain AarI and EcoRV restriction sites adjoining the exon 2, and an additional AarI site directly after the exon 3.

The left homologous arm (LHA) was PCR amplified with the LHAdGαo23fw and LHAdGαo23rev primers from genomic DNA using Phusion High-Fidelity DNA Polymerase (New England Biolabs, Ipswich USA, cat. #M0530S), producing a 1060bp PCR product, which was further cloned into the pUC57-dhGαo23 plasmid by the EcoRV site producing the construct pLdhGαo23. The right homologous arm (RHA) was PCR amplified with the RHAdGαo23fw and RHAdGαo23rev primers, and the resulting 1070 bp PCR product was cloned into the plasmid pHD-ScarlessDsRed (Drosophila Genomics Resource Center, Bloomington USA, stock #1364) into the SapI site, producing the construct pScarless-dhGαoR. Both cloning steps were performed with the NEBuilder HiFi DNA Assembly Cloning Kit (New England Biolabs, cat. #E5520S). The AarI-AarI fragment from pLdhGαo23 was cloned into the AarI site of pScarless-dhGαoR. The resultant donor plasmid pLdhGαo23R contains humanized exons 2 and 3 of *Gαo* flanked with the 1000 bp-long LHA and RHA. The end of exon 3 of *Gαo* in the plasmid is modified by insertion of the piggyBac transposon between the duplicated TTAA sequence. The precise excision of the piggyBac restores the correct exon sequence with unique TTAA.

#### 2.1.2. Donor Plasmid for the Second Round of Humanization dGαo

The DNA fragment of humanized *Drosophila Gαo* containing exons 4 to 7 with introns between them and 120 bp intronic flanking sequences before exon 4 and after exon 7 was synthesized by Synbio Technologies as the plasmid pUC57-dhGαo47. pUC57-dhGαo47 was used as a template for the PCR amplification of two fragments: one with the primer set LHAdGαo47fw/LHAdGαo47rev and the other with the primer set RHAdGαo47fw/RHAdGαo47rev. The 650 bp and 430 bp PCR products amplified, respectively, were mixed with pHD-ScarlessDsRed pre-digested with SapI (New England Biolabs, cat #R0569S) and AarI (Thermo Fisher Scientific, Waltham. MA, USA, cat. #ER1581) restriction enzymes and circulated using the NEBuilder HiFi DNA Assembly Cloning Kit. The resultant donor plasmid pLdhGαo47R contains humanized exons 4–7 of *Gαo* flanked with the short 130 bp LHA and RHA and the piggyBac transposon marked with 3xP3-DsRed inserted into the duplicated TTAA sequence in the exon 6.

#### 2.1.3. Plasmids Providing Expression of gRNAs under the Control of the Drosophila U6:3 Promoter

CRISPR targets sites were identified using Target Finder [55], targetfinder.flycrispr.neuro.brown.edu/.

Complimentary oligonucleotides GTCGGACTTTAAACAATATCGAC and AAACGTCGATATTGTTTAAAGTC, GTCGGCCAGCAGCTCCTCCGAGA and AAACTCTCGGAGGAGCTGCTGGC, GTCGTGGCAGGACGCCGGTGTCC and AAACGGACACCGGCGTCCTGCCA, GTC**G**GCAAACAACCTGCGCGGCTG and AAACCAGCCGCGCAGGTTGTTTGC, GTC**G**GACCACTCACCTGTGTATT and AAACAATACACAGGTGAGTGGTC, GTC**G**TTTCCTGGACGATTTGGAT and AAACATCCAAATCGTCCAGGAAA were annealed and cloned into pCFD3-dU6:3gRNA (Addgene, Watertown USA, cat. #49410), which was digested with BbsI (New England Biolabs, cat. #R0539S). A total of 6 plasmids from pCFD-gRNA1 to pCFD-gRNA6 were constructed. Set plasmids pCFD-gRNA1, pCFD-gRNA2, pCFD-gRNA3 combined with the donor plasmid pLdhGαo23R were used for the first round of transgenesis; pCFD-gRNA4, pCFD-gRNA5, pCFD-gRNA6 together with the donor plasmid hdGαo47ScarlessDsRed—for the second one.

### 2.2. Flies, Germline Transformation

Flies were maintained at 25 °C on the standard medium. The strain y[1] sc[*] v[1] sev[21]; P{y[+t7.7] v[+t1.8]=nos-Cas9.R}attP2 expressing Cas9 in the germline under the control of the *nos* promoter (Bloomington Drosophila Stock Center (BDSC, Bloomington USA), stock #78782) was used for germline transformation in the first round of transgenesis. The resultant fly stock *Gαo[h23ex4aa-1]* was combined with *P{y[+t7.7] v[+t1.8]=nos-Cas9.R}attP2* and used for the second round of transgenesis. The strain *w[1118]; In(2LR)Gla, wg[Gla-1]/CyO; Herm{3xP3-ECFP,alphatub-piggyBacK10}M10* expressing PiggyBac transposase (BDSC, stock # 32073) was used for the excision of the PiggyBac-based marker. The resultant alleles and their derivatives were balanced over *CyO*.

Germline transformation was performed as described in the Gompel’s lab protocol (*Drosophila* germline transformation. Available online: gompel.org/wp-content/uploads/2015/12/Drosophila-transformation-with-chorion.pdf (accessed on 05.10.2020)). Embryos were injected with the donor plasmid (500 ng/μL) and three gRNA plasmids (100 ng/μL each).

Transformants were selected under a fluorescence stereomicroscope (Zeiss SteReEO Discovery.V8, Carl Zeiss, Jena Germany) using the Filter Set 43 HE for DsRed fluorescent dye detection (excitation BP 550/25; emission BP 605/70).

### 2.3. Molecular Analysis

**Figure 2:**
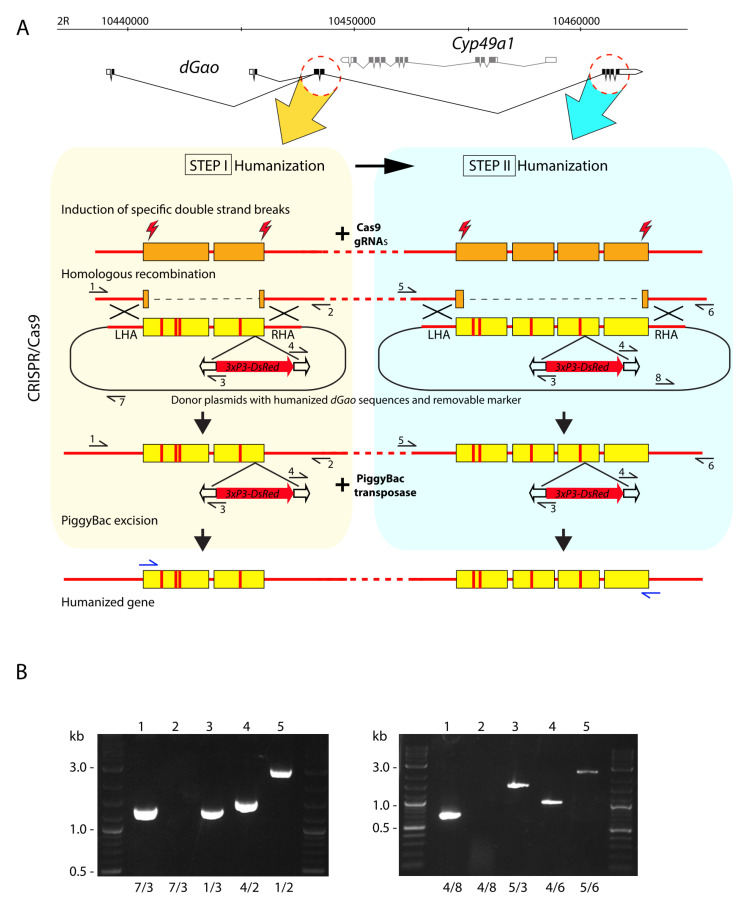
(**A**) Two-step strategy for genomic humanization of dGαo. The primers for validation of the correct donor sequence integration in the genome are designated with black horizontal arrows and marked with digits. Primers used in RT-PCR are designated with blue horizontal arrows. LHA and RHA: left and right homologous arms in the donor plasmids required for homologous recombination with the genomic sequences. The exons with vertical red lines schematically designate humanized sequences with mismatches. (**B**) Agarose gels with PCR products obtained with the primers as numbered below each lane (see Supplementary Table S1 for sequences). Lanes 1: control PCR from donor plasmids pLdhGαo23R (left panel) and pLdhGαo47R (right panel). The absence of a PCR product (lanes 2) performed with the same primers from the genomic DNA of transgenic flies indicates the correct integration of the donor sequences without the plasmid backbone. Lanes 3 and 4: verification of the entire integration of the donor sequence in the genome before excision of the 3xP3-dsRed marker. Lanes 5: PCR through the integrated donor sequence after the excision of the 3xP3-dsRed marker from genomic DNA of the homozygous stocks *Gαo[h23ex4aa-1]* (left panel) and *Gαo[humanized-1]* (right panel).

Genomic DNA was isolated from individual flies of different genotypes as described previously [56]. PCR analysis was carried out with different primer sets (Supplementary Table S1, Figure 2B) using Phusion High-Fidelity DNA Polymerase following the manufacturer’s instructions.

Total RNA was isolated with the NucleoSpin RNA kit (Macherey-Nagel, Dueren Germany) from 30 adult flies for each sample. cDNA was synthesized by priming with oligo-dT with RevertAid Reverse Transcriptase (Thermo Fisher Scientific, cat. #EP0441) following the manufacturer’s instructions. The 958 bp PCR products including the region from exon 2 to exon 7 were amplified from cDNA with the primers dGαomRNAfw and dGαomRNArev.

### 2.4. Immunochemistry and Microscopy

The following primary antibodies were used: mouse monoclonal antibody against bovine Gαo (1:20 dilution, Santa Cruz Biotechnology, Dallas USA, cat. #sc-13532) and rabbit anti-dGαo (1:50; [40]). Secondary antibodies were donkey anti-mouse Cy3 and donkey anti-rabbit 488 (Jackson ImmunoResearch, West Grove USA, cat. #715-165-150 and #711-545-152), used at the 1:300 dilution.

Ventral nerve cords and brains from third instar larvae were fixed with 4% paraformaldehyde in PBS, permeabilized in 0.5% NP-40 and immunostained in 0.2% Tween20 in PBS. Coverslips were finally mounted with Vectashield (Vector Labs, Burlingame USA, cat. #H-1000-10) for microscopy analysis. Humanized and wild-type (control) larval tissues were stained simultaneously in the same vial in order to control the immunostaining specificity.

Fluorescent images were acquired with a Zeiss LSM 800 Airyscan confocal microscope and the images were reconstructed from Z-stacks using ZEN blue software (Carl Zeiss). All images were processed using the same confocal settings.

### 2.5. Behavioural Assays

The negative geotaxis assay was performed as described [57]. In brief, 2-to-5-day-old flies, in groups of 15, males and females separately, were placed in empty polystyrene vials. Post gentle tapping was used to bring the flies to the vial bottom, the vial was placed vertically, and the flies climbing above the 8 cm distance in 10 s were counted, producing the climbing pass rate as the percent of total flies. Ten groups were tested for each genotype, each group was tested three times. The aversive phototaxic suppression assay, based on the ability of flies to memorize the association of light stimulus with aversive odour (quinine hydrochloride), was performed as described [57].

## 3. Results and Discussion

Comparative analysis of Gαo homologs reveals a high degree of similarity in the amino acid sequences of proteins as well as in the exon-intron structures of the genes encoding them. Even evolutionary distant organisms retain the same nucleotide length of the coding sequences in alternatively spliced transcripts and have the resultingly fixed protein length: 354 aa. However, lengths of the genomic loci vary in a wide range from species to species. In humans, the respective locus extends for 165 kb, in *Drosophila*—for 28 kb, and in *C. elegans*—for 4.5 kb (see the gene links: NCBI, *GNAO1*. Available online: ncbi.nlm.nih.gov/gene/?term=2775 (accessed on 05.10.2020); FlyBase, *dGao*. Available online: flybase.org/reports/FBgn0001122 (accessed on 05.10.2020 (also see Figure 2A) and WormBase, *goa-1.* Available online: wormbase.org/species/c_elegans/gene/WBGene00001648#0-9f-10, respectively, Supplementary Figure S1). Interestingly, all of them have very similar sets of coding exons (Figure 1B). For example, 191 amino acids between 101aa and 292aa are coded by four exons with the lengths 161, 129, 130 and 154 bp in both invertebrates and humans. The percent of similarity between human and *C. elegans* Gαo proteins is 86.7%, with 82.5% identity. The *Drosophila* protein is slightly more similar to the human one: 86.5% similarity and 83.9% identity, with only 57 mismatching amino acids out of 354. The alignment of five protein sequences (two highly similar Gαo isoforms are encoded from the same gene both in humans and in *Drosophila*) reveals the presence of conservative and independently evolving blocks, which have not changed through hundreds of millions of years (Figure 1B).

With this level of conservation, it is perhaps not surprising that of the de novo point mutations so far identified in patients suffering from *GNAO1* encephalopathies (altogether 34 different point mutations occurring in 27 sites), all but one fall into amino acids identical between human, *Drosophila* and nematode Gαo sequences (Figure 1A). This fact suggests that these amino acids play instrumental functions for the activity of Gαo and also provides the ground to attempt the modeling of *GNAO1* encephalopathy in *Drosophila*. For the sake of terminology, we will refer to the *Drosophila* and human genes as *Gαo* and *GNAO1*, and to the proteins they encode as dGαo and hGαo, respectively.

*Drosophila Gαo* produces two variants of the protein through alternative splicing of the first coding sequence-containing exons. The six downstream exons are common for both transcripts (Figure 1B). These splice variants differ only in seven amino acids, making them 98% identical to each other. In a pairwise comparison with the two isoforms, hGαo is closer to the dGαo-B isoform (Supplementary Figure S2). Both variants of dGαo are expressed at similar levels during the life cycle, with one minor exception: one of the isoforms starts to be transcribed in very early embryos and the other one—10 h later [36]. The two alternatively spliced first coding exons are distanced from each other by 6kb. The six downstream common exons are grouped into two clusters with 2 and 4 exons, which are separated by a long intron containing embedded gene *Cyp49a1*, oriented in the direction opposite to *Gαo* (Figure 2).

The locus of human *GNAO1* contains a duplication of the two last exons, which are spliced alternatively to produce two protein isoforms (hGαoA and hGαoB) with amino acids variations in the C-terminus. In a pairwise comparison with the two human isoforms, dGαo is closer to the hGαoB isoform (Supplementary Figure S2). Unlike the two *Drosophila* isoforms, the two human splice variants of *GNAO1* are expressed differently, with hGαoA being the major version expressed [58]. All known mutations (from E246K to Y291N) (Figure 1A) in the alternatively spliced exons 7 and 8 belong to hGαoA. Thus, we concentrated on this variant in our replacement strategy.

In order to preserve the endogenous structure of the *Gαo* locus with its potential regulatory elements such as enhancers, promoters, insulators, etc., we developed the strategy of sequential *Gαo* editing during the humanization process (Figure 2A). In order to ensure the non-damaged *Gαo* transcription start sites, correct splicing with production of the two *Gαo* transcripts with endogenous and undamaged 5’UTR sequencing, this strategy involved keeping untouched the non-coding exon together with the first coding exon, which is subject to the alternative splicing. The sequences of the core of the gene were humanized in two steps. In the first step, we humanized the exons 2 and 3; in the second—exons 4 to 7 (Figure 2A). Both rounds of transgenesis were performed with the CRISPR-Cas9 technology (see Methods for details). As a template for the homologous recombination, we used donor plasmids containing *Drosophila* DNA sequences, whereas the codons different between *Gαo* and *GNAO1* contained the minimally needed nucleotide substitutions ensuring encoding of the human amino acids. Additionally, the donor plasmid contained synonymous nucleotide substitutions in the sequences corresponding to the Cas9/gRNA complex targets preventing their recognition and destruction by the complex.

For the gene editing, we used the transgenic fly strain expressing Cas9 in the germline. In order to replace the first cluster of exons, we injected embryos with the mixture of four plasmids: the donor plasmid for homologous recombination and three plasmids producing the gRNAs for the induction of breaks in the target genome locus (see Methods). Besides the modified *Gαo* exons and the homologous arms (LHA and RHA), the donor plasmids contained a 3xP3-DsRed marker cassette flanked by the PiggyBac transposon ends inserted in the end of the 3^rd^ exon. Transgenic flies were selected by red fluorescence in eyes provided by the expression of DsRed under the eye-specific 3xP3 promoter [59]. The established transgenic fly strains were verified by PCR with the primers annealing to the neighbouring genomic sequences outside of the homologous arms and inside the PiggyBac transposon. The correct sizes of the PCR products indicated the proper integration of the donor plasmid (Figure 2B). The fly strains with integration of the whole donor plasmid were excluded from the analyses by using primers annealing at the body of plasmid. About one third of the transgenic lines contained the plasmid integrated via the rolling circle replication mechanism and thus had to be discarded.

Three independent transgenic lines with the full set of substitutions, and two independent transgenic lines lacking 4aa substitutions in the 2^nd^ exon (see below for the explanation on the 4 aa) were identified after the sequencing analysis of the PCR products and selected for further propagation. These fly lines were crossed with the strain encoding the PiggyBac transposase. The excision of the marker gene was identified in the second generation by the loss of red fluorescence in eyes. Since the mobilization of PiggyBac-based transposons is characterized by precise excisions without indels in the insertion site, the integrity of the 3^rd^ exon was restored and, consequently, this partially humanized gene started producing proper transcripts. Finally, three independent strains with the full set of substitutions in the first exon cluster, along with the two independent strains without 4aa substitutions in the 2^nd^ exon, were established. These alleles were named *Gαo[h23ex-1]*, *Gαo[h23ex-2]*, *Gαo[h23ex-3]*, and *Gαo[h23ex4aa-1]*, *Gαo[h23ex4aa-2]*, respectively. Both *Gαo[h23ex4aa]* lines demonstrated clear viability and fertility in homozygosity. In a sharp and curious contrast, all the three *Gαo[h23ex]* lines were homozygous lethal.

After removing the marker gene, both allele types were confirmed by PCR and sequencing. Then, we extracted total RNA from adult flies, and *Gαo* transcripts were examined through RT-PCR using the primers for the wild-type and mutant alleles. The PCR products amplifying the 958 bp region between the exons 2 and 7 exons were verified by sequencing, revealing that all the mutant transcripts were spliced correctly, and, as expected, that both the wild-type and the mutant transcripts were present in the heterozygous *Gαo[h23ex]* stocks. Homozygous *Gαo[h23ex4aa]* stocks produced mutant transcripts only. Due to the unexplained lethality of the *Gαo[h23ex]* flies, the second round of humanization was performed on the *Gαo[h23ex4aa-1]* strain.

The second cluster was edited in the same manner as the first one: the *Gαo[h23ex4aa-1]* stock combined with the Cas9-expressing transgene was used for the germline transformation (see Methods). Transgenic flies were selected by the fluorescent marker, and then verified by PCR (Figure 2B) followed by sequencing both before and after the excision of the marker; ultimately, the resultant transcript sequences were also verified. Two independent fly lines obtained through the two-step humanization process were established. In these lines, 26 out of the 27 amino acids different between the human and *Drosophila* Gαo were replaced (except of F156Y, apparently not substituted as a result of gRNA imperfection, adding this non-replaced amino acid to the four not replaced in the first round of humanization). These alleles were named *Gαo[humanized-1]* and *Gαo[humanized-2]*. Both lines were homozygous viable and fertile.

As a result of this two-step CRISPR/Cas9-mediated humanization, we succeeded to replace, in the endogenous *Gαo* locus, 49 amino acids of dGαo with the human Gαo sequences. The resultant identity between hGαo and the humanized *Gαo* isoform dGαo-A reached 96.8%, and the humanized isoform dGαo-B—97.6%. These values are similar to those between the mouse and human Gαo sequences and are much higher than within any vertebrate-invertebrate Gαo pair.

**Figure 3:**
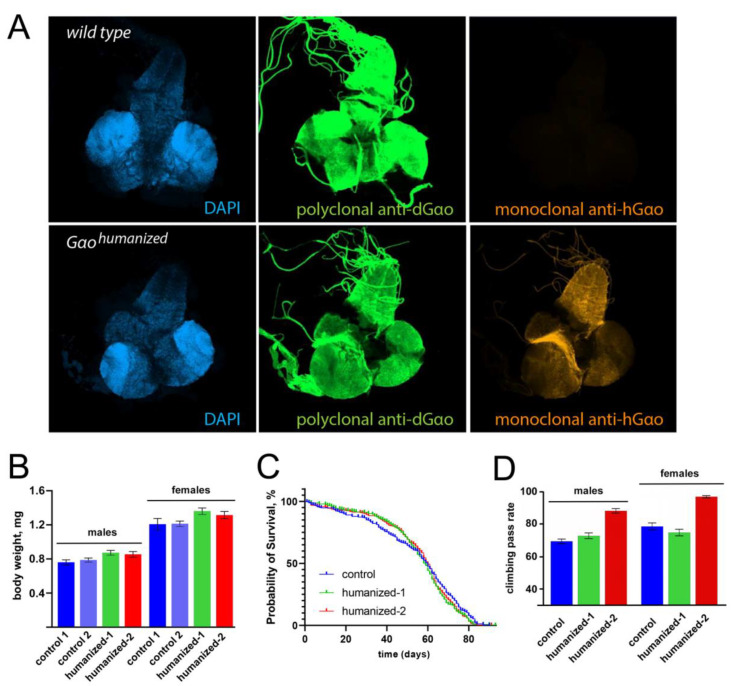
(**A**) Expression of humanized and wild-type Gαo in brains and ventral nerve cords of 3rd instar larvae. The upper panel represents a sample from wild-type larvae and the bottom panel—from *dGao[humanized]*. The immunostaining demonstrates specific recognition of the humanized protein (bottom right) with the monoclonal antibody recognizing hGαo; the polyclonal anti-dGαo antibodies, in contrast, recognize both dGαo and hGαo (the latter with a lower affinity as seen in the middle panels, which were recorded with identical confocal settings). (**B**) The body weight of the humanized flies is normal. The body weight in two control lines (the line used for initial injection is control 1 and an independent line is control 2) and the two homozygous humanized lines generated in our study was determined through measuring the weight of groups of fifty adult (2–3 days after hatching) male or female flies kept under non-crowding conditions; the individual fly body weight was then recalculated. Data are shown as mean ± sd, *n* = 11 to 13 groups of fifty flies. (**C**) The life span of 250 adult flies (125 males and 125 females; control animals were of the same genotype as ‘control 1’ in (**B**)) was determined during 93 days. (**D**) The locomotion behaviour of adult flies was measured in the negative geotaxis assay. Ten groups of 15 flies were measured three times for males, and the same for females, for each genotype. Data are shown as mean ± sem, *n* = 30. The T-test shows significant differences between the *Gαo[humanized-2]* line and the control line, as well as between the *Gαo[humanized-2]* line and the *Gαo[humanized-1]* line, for both males and females (*p*-values < 10^−8^), but not between the *Gαo[humanized-1]* and control lines (*p*-value > 0.1).

In addition to the validation of the humanized transcript expression (Figure 2B), we confirmed hGαo expression in the *Drosophila* central nervous system by immunostaining. Brains together with ventral nerve cords from third instar larvae were probed with antibodies against dGαo (polyclonal) and hGαo (monoclonal, see Methods). We found that the polyclonal antibodies expectedly recognized both *Drosophila* and human proteins in our samples, while the monoclonal antibodies had affinity exclusively to the human Gαo (Figure 3A). The pattern of immunostaining with different antibodies was identical for both genotypes. These findings support our conclusion that hGαo is expressed in a proper way under the endogenous *Gαo* gene regulatory elements. This expression of hGαo was sufficient to fully recapitulate the endogenous functions of dGαo, as judged by the fertility of the humanized lines and their full viability, measured, e.g., by their normal body weight (Figure 3B) and life span (Figure 3C).

To further verify the completeness of the ability of the humanized Gαo to recapitulate the endogenous neuronal dGαo functions, we analysed the locomotion and memory performance of the humanized lines. As shown in Figure 3D, the locomotion measured in the negative geotaxis assay [57] revealed equivalent performance of the control and *Gαo[humanized-1]* lines. Curiously, the *Gαo[humanized-2]* line displayed an increased locomotion in the assay, both in comparison to the control and *Gαo[humanized-1]* line (Figure 3D). While we are currently unable to explain the better performance of this second humanized line, we conclude that the humanization of dGαo at least does not impede the locomotor behaviour of the fruit flies, indicating that the sophisticated brain and neuromuscular circuitry needed for the locomotion is functional in the humanized flies. Furthermore, the humanized flies revealed the ability to memorize the association of light (which is normally attractive to *Drosophila*) with an adverse odour (quinine) in the aversive phototaxic suppression assay [57], proving competence in vision, olfaction, locomotion, learning and memory (M. Savitsky. University of Geneva, 1211 Geneva, Switzerland. Preliminary data). These complex behavioural competences will be important in the modeling of *GNAO1* encephalopathy in the future studies.

The replacement of a model organism’s protein-coding sequence with its human ortholog, preserving the endogenous gene expression regulators, represents an important tool to investigate the evolutionary and functional conservation of the gene of interest; this approach is particularly important for pathology-related genes [60]. A systematic investigation into the efficiency of gene humanization has been performed in yeast [61]. Despite several successful investigations in *Drosophila* [62,63], the fruit fly has not been systematically studied in this regard. Our study describes the first ever replacement of an endogenous *Drosophila* G protein with the orthologous human sequence. We find it remarkable that humanized Gαo fully recapitulates the numerous *Drosophila* protein functions, resulting in phenotype-less, viable and fertile flies, having the normal life cycle, locomotory activity, and longevity. The resultant *Drosophila* strain humanized for the *Gαo* gene can now be used to incorporate human mutations found in *GNAO1* encephalopathy patients.

*GNAO1* encephalopathies belong to the group of paediatric syndromes collectively referred to as developmental and epileptic encephalopathies (DEEs). Multiple DEEs have been modelled in *Drosophila* [54]. Of the genes implicated in human DEE, mutations in their *Drosophila* orthologues produce seizures or paralysis, as described, e.g., for the voltage-gated potassium channels Kv1.2 (*Shaker* in flies and *KCNA2* in humans [64,65]) and Kv2.1 (*Shaker cognate b* in flies and *KCNB1* in humans [66,67]), the voltage-gated sodium channel (*paralysis* in flies and *SCN1A* in humans [68,69]), or the synaptic vesicle endocytosis regulator dynamin (*shibire* in flies and *DNM1* in humans [70,71]). The successful establishment of a *Drosophila* model of a DEE can then be followed by two types of translational investigations: a suppressor-enhancer screening and a drug candidate screening. The first brings knowledge on the molecular partners of a disease protein, some of which may emerge as promising drug targets to ultimately develop a treatment of the human DEE. As an example, a suppressor-enhancer screening of the *paralysis* mutant identified *gilgamesh* to modify the manifestation of *paralysis* seizures; the human orthologue of *gilgamesh* is casein kinase gamma3, emerging as a potential new target to develop seizure therapeutics [72]. As a proof-of-concept for the second, several *Drosophila* epileptic mutants, including *paralysis*, showed amelioration of the phenotypes upon treatment with anticonvulsants such as gabapentin used to treat human epilepsy patients [73].

Despite the power of *Drosophila* genetics to model DEE and other neurological and non-neurological human diseases, to dissect the molecular aetiology of the disease and to screen for drug candidates [74], every model has its limitations. After all, *Drosophila* has significantly fewer neurons than human patients, and fewer genes as well. Although around 75% of pathology-related human genes have orthologues in *Drosophila* [75], the proteins they encode are not identical, which may constrain the way discoveries (especially discoveries of drug candidates) to be made on the *Drosophila* model can be translated to the human conditions. In this regard, humanization of the fruit fly orthologue prior to the establishment of the disease model can be viewed as a risk mitigation strategy.

Our work describes the first essential step towards the establishment of a *Drosophila* model of *GNAO1* encephalopathies. The full power of *Drosophila* genetics and drug discovery tools available in this model organism [74,76] will then become accessible to uncover the molecular mechanisms of the aetiology of *GNAO1* encephalopathy and to provide potential therapeutic leads to treat it. The progressive manner of this devastating disease affecting infants and the current lack of efficient treatment makes this approach highly desired.

## Supplementary Materials

The following are available online at https://www.mdpi.com/2227-9059/8/10/395/s1.
