# Supplementary material for: Humanization of Drosophila Gαo to Model GNAO1 Paediatric Encephalopathies
Source: Biomedicines. 2020 Oct 6;8(10):395. doi: 10.3390/biomedicines8100395 (PMC7599900; doi:10.3390/biomedicines8100395)
Supplement: Supplementary file 1 [file biomedicines-08-00395-s001.pdf]

D.melanogaster Gαo

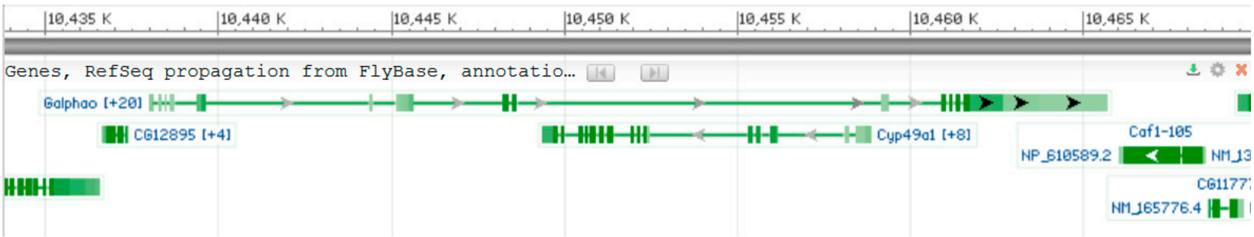

C.elegans goa-1

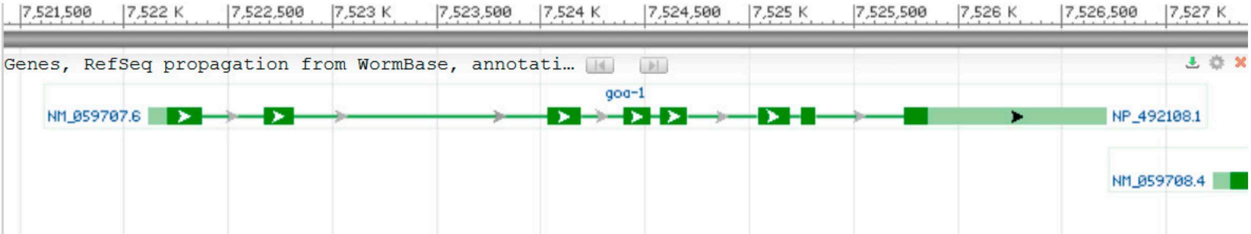

H.sapiens GNAO1

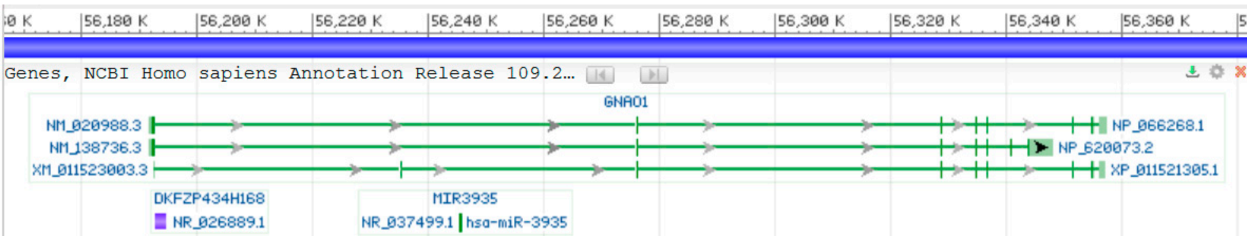

Supplementary Figure S1. Loci of *Gαo* genes have variable size but similar exon-intron structures (see also Fig. 1B). NCBI Gene ID: 36104 (primary source FLYBASE:FBgn0001122) for *D. melanogaster*, 172505 (primary source WormBase:WBGene00001648) for *C. elegans*; 2775 (primary source HGNC:4389) for *H. sapiens.*

A

```
dGao-PA     1 MGCAQSAEERAAAARSRLIERNLKEDGIQAAKDIKLLLLG
dGao-PB     1 MGCTTSAEERAAIQRSKQIEKNLKEDGIQAAKDIKLLLLG
hGao        1 MGCTLSAEERAALERSKAIEKNLKEDGISAAKDVKLLLLG
                ***..*******..**..**.*******.****.******
```

B

```
hGαoA        242 NRMHESLMLFDSICNNKFFIDTSIILFLNKKDLFGEKIKKSPLTICFPEYTGPNTYEDAA
dGαo         242 NRMQESLKLFDSICNNKWFTDTSIILFLNKKDLFEEKIRKSPLTICFPEYTGGQEYGEAA
hGαoB        242 NRMHESLKLFDSICNNKWFTDTSIILFLNKKDIFEEKIKKSPLTICFPEYTGPSAFTEAV

hGαoA        302 AYIQAQFESKNRSPNKEIYCHMTCATDTNNIQVVFDAVTDIIIANNLRGCGLY
dGαo         302 AYIQAQFEAKNKSTSKEIYCHMTCATDTNNIQFVFDAVTDVIIANNLRGCGLY
hGαoB        302 AYIQAQYESKNKSAHKEIYTHVTCATDTNNIQFVFDAVTDVIIAKNLRGCGLY
```

Supplementary Figure S2. A. Alignment of Gαo proteins (amino acids are coded by 1st exons). *D. melanogaster* has splice variants with variable 1st coding sequence-containing exons. Most non-conservative amino acids between two Drosophila isoforms overlap non-conservative amino-acids between Drosophila and human. B. Alignment of Gαo proteins (amino acids are coded by two last exons). *Human* has splice variants with variable two last exons. hGαoA (settled as reference sequences here) has more identity with Drosophila's sequences than with hGαoB (17 mismatches vs 20). There are 16 mismatches between dGαo and hGαoB.

Supplementary Table S1. Sequence of primers used in the molecular analysis and cloning.

Primer	Sequence
dGaomRNAfw	GGTGAGTCGGGCAAGAGCACAATA
dGaomRNArev	GGCCTGGAATCCATCTTAGTACAGTCCA
LHAdGao23fw	cacctgcgaatccgatTGTGGACTTTTTCAAGTGGTGGA
LHAdGao23rev	GAAGCCGTCCTCGTGAATGATTTTC
RHAdGao23fw	gactatctttctAGGGTTAACGATTCCGCAAAATAGTAAGTACCAAATCA
RHAdGao23rev	atggtcttcttttcCCGGAAACTAACGCTGAGGGACGAGTG
LdGao23fw (1)	CGCTGCTGCTCTTGAGTTTTCCA
RdGao23rev (2)	TCGTGAGTTTGCCCTTTGGCTTT
LHAdGao47fw	tagtgtcttcggggccGAAAATTGAGAATGGACGGGTGGA
LHAdGao47rev	ttatctttctagggTTAATCTTCTCCCCGAACAAATCCT
RHAdGao47fw	ctatctttctagggTTAAGAAGAGTCCCCTGACGATT
RHAdGao47rev	atggtcttcttttcccGGTTACGGTGTTCCCTGCTAA
LdGao47fw (5)	AAGCTGTTTGCATAGCCAAGTGAG
RdGao47rev (6)	ACAACTGTATGCAATGTTGGCGCTTGTG
pUC-L (7)	GCGCCTGTCACTTTGCTTGATA
pUC-R (8)	CGATGGTAGTGTGGGGACTCC
pBac_rev (4)	GAGAGAGCAATATTTCAAGAATGC
pBacWTlong (3)	CCGATAAAACACATGCGTCAATTT

Lowercase letters designate overlap sequences, required for the assembly of adjacent fragments upon cloning using the NEBuilder HiFi DNA Assembly Cloning Kit (New England Biolabs). Numbers in brackets after the primers' names represent how they are designated in Fig. 2B.
